# Vitamin D Status at Admission and Its Association With Mortality in Children Admitted to the Pediatric Intensive Care Unit

**DOI:** 10.7759/cureus.8413

**Published:** 2020-06-02

**Authors:** Kiran kumar M, Sarthak Das, Niranjan Biswal, Narayanan Parameswaran, Nivedita Nanda

**Affiliations:** 1 Paediatrics, Jawaharlal Institute of Postgraduate Medical Education and Research, Puducherry, IND; 2 Paediatrics, All India Institute of Medical Sciences (AIIMS), Mangalagiri, IND; 3 Biochemistry, Jawaharlal Institute of Postgraduate Medical Education and Research, Puducherry, IND

**Keywords:** children, mortality, paediatric intensive care unit, sepsis, vitamin d

## Abstract

Introduction

This study aims to evaluate the prevalence of vitamin D deficiency and the correlation between serum 25-hydroxyvitamin D (25(OH)D) and mortality.

Materials and methods

A prospective observational study was conducted among 522 children admitted to the Pediatric Intensive Care Unit in the Pediatrics Department of the Jawaharlal Institute of Postgraduate Medical Education and Research, Pondicherry, India. After measuring vitamin D levels, children were assigned into three groups based on their serum 25(OH)D levels: a sufficient group (25(OH)D level ≥ 30 ng/mL), an insufficient group (25(OH)D level = 20-29.9 ng/mL), and a deficient group (25(OH)D level < 20 ng/mL). Each group was again divided into two sub-groups (survivors and non-survivors if death was the outcome), and then each sub-group was again divided into two groups (sepsis and all non-septic causes). Results were evaluated for an association with mortality.

Results

A majority (66.6%) of patients who died had low levels of 25(OH)D (deficient = 37.9%; insufficient = 28.7%). Mortality was higher in children with 25(OH)D deficiency (P = 0.03). In univariate analysis, 25(OH)D deficiency was strongly associated with sepsis in children according to our observation, with 51% from the deficient group, 38% from the insufficient group, and 27.5% from the sufficient group (P ≤ 0.01). Mortality is not associated with 25(OH)D deficiency or insufficiency in multilogistic regression analysis. A serum vitamin D level of 20 ng/mL can predict higher mortality, with a specificity of 62.1%.

Conclusions

Vitamin D supplementation may be recommended for PICU-admitted cases to decrease the risk of sepsis. This association can be explored more in the future among the community population for further recommendations.

## Introduction

Vitamin D is essential for the growth and maturation of the skeletal system. However, many recent studies showed that vitamin D also plays a significant role in immunity, and its deficiency is associated with infections [1]. Vitamin D is associated with anti-inflammatory and anti-proliferative mechanisms, and its deficiency may result in an increased risk of mortality in malignancy, cardiovascular, and autoimmune diseases [2]. The above actions are carried out by vitamin D receptors present in bone, intestine, and growth plate [3].

There is increasing evidence of the association of vitamin D deficiency with adverse outcomes like a prolonged hospital stay, increased infection rates, and increased mortality. The highest percent of mortality in pediatric intensive care units (PICUs) was associated with sepsis and infective causes. Also, severe sepsis, sepsis-related mortality, and an overall increase in mortality in both adult intensive care units and PICUs have been reported with vitamin D deficiency [4-6].

Even though there are many studies in other countries, reports regarding vitamin D deficiency and its association with adverse outcomes in an Indian scenario are scant [7,8]. Indian data have shown no significant association with clinically relevant outcomes [7,8]. Therefore, we conducted a prospective observational study of the association between vitamin D deficiency and mortality, especially with sepsis, among patients admitted to the PICU.

## Materials and methods

This study was a prospective observational study involving children admitted to the PICU from November 2014 to October 2015 in the Pediatrics Department of the Jawaharlal Institute of Postgraduate Medical Education and Research, Puducherry, India. All PICU-admitted cases of aged 1 month to 12 years were included in the study. Known cases of rickets or patients who were diagnosed with rickets for the first time after hospital admission, those who received vitamin D supplementation within 30 days of admission, and those admitted for post-procedure monitoring, snake bite, and post-operative monitoring in the PICU were excluded from the study.

After obtaining informed consent from parents and guardians, children were enrolled in the study. Parents and guardians were questioned via proforma, and information was collected from the parents regarding the child's health status, duration of illness, exposure to sunlight by play or outdoor activities, and intake of vitamin supplements.

Blood was collected as early as possible by drawing 2 mL of venous blood from children within 24 hours of admission to the PICU. After collection, blood was allowed to clot at room temperature, and serum was separated by centrifugation. Collected serum was frozen at -80°C, stored, and then processed. An enzyme-linked immunosorbent assay developed by Calbiotech (El Cajon, CA, USA) and procured by BioDiagnostics (Longmont, CO, USA) was used for measuring 25-hydroxyvitamin D (25(OH)D) levels.

After collecting patient data and measuring vitamin D levels, children were assigned into three groups based on their serum 25(OH)D levels: a sufficient group (25(OH)D level ≥ 30 ng/mL), an insufficient group (25(OH)D level = 20-29.9 ng/mL), and a deficient group (25(OH)D level < 20 ng/mL) [9].

**Figure 1:**
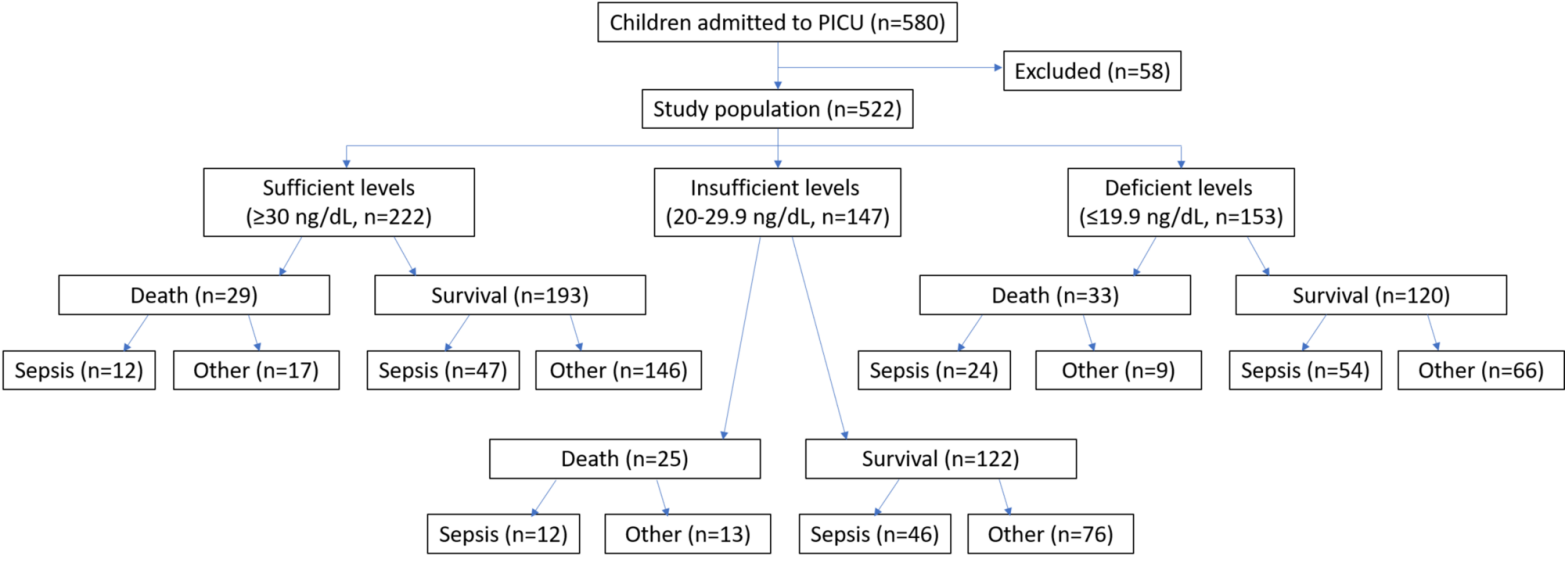
Study design flow chart. PICU, pediatric intensive care unit

Each group was again divided into two sub-groups (survivors and non-survivors if death was the outcome) and then each sub-group was again divided into two groups (septic and non-septic causes). Culture positivity from any body fluids or serological evidence (C-reactive protein, procalcitonin) or increased total leukocyte count or neutrophilic leukocytosis performed during PICU stay qualified them for either the septic or non-septic groups. Suspected sepsis included cases satisfying systemic inflammatory response syndrome or meeting community-acquired pneumonia with negative microbial testing and response to antibiotic therapy. Septic shock requiring vasopressor therapy during hospital stay was also included in the sepsis group (Figure 1) [4].

Statistical analysis

Data were statistically presented as mean ± SD, frequencies (number of cases), and percentages when appropriate. Data were tested first for normal distribution using the Kolmogorov-Smirnov test. A comparison of quantitative variables between the study groups was made using Student's t-test for independent samples, if normally distributed. The Mann-Whitney U test was used for non-normally distributed quantitative data. Comparison for more than two groups was made by one-way analysis of variance with post-hoc Tukey's test. For comparing categorical data, a chi-square test was used. Fisher’s exact test was used, instead, for all 2 x 2 tables.

Receiver operating characteristic (ROC) curves were generated to find the efficacy of vitamin D levels as screening tests for predicting survival. Binary logistic regression analysis was also performed to find the significant predictors of the outcome by taking survival as the dependent variable, and variables such as serum 25(OH)D levels, sepsis, protein energy malnutrition, and culture positivity as independent variables.

All statistical calculations were performed using computer programs Microsoft Excel 2007 (Microsoft Corporation, New York, NY, USA) and SPSS Statistics for Windows, Version 17.0 (SPSS Inc., Chicago, IL, USA). A probability value (P-value) of less than 0.05 was considered as statistically significant.

## Results

**Figure 2:**
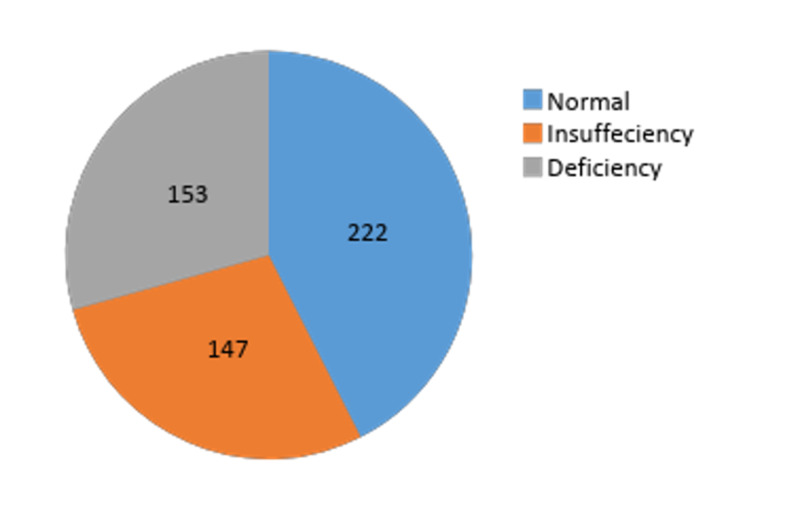
Proportion of 25(OH)D levels.

Of the 580 total patients admitted to the PICU, 522 patients were included in the study. Among these, 25(OH)D deficiency was observed in 29.3%, insufficiency in 28.2%, and sufficient in 42.5% children (Figure 2). The median serum 25(OH)D level was 13.80 ng/mL (interquartile range [IQR] 10.08-17.69) in the deficient group, 24.68 ng/mL (IQR: 22.28-27.37) in the insufficient group, and 39.62 ng/mL (IQR: 33.8-54.28) in the sufficient group.

The baseline characteristics of the study population are presented in Table 1. Of the 522 children in the study, 54.6% (n = 285) were boys and 45.40% (n = 237) were girls. Among all boys, 59.5% (n = 91) children were in the deficient group, 55.1% (n = 81) in the insufficient group, and 50.9% (n = 81) in the sufficient group (p = 0.3).

**Table 1:** Baseline characteristics of the study population

Baseline characteristics	25(OH)D status				
Age group	Sufficient, n (%)	Insufficient, n (%)	Deficient, n (%)	Total	P-value
Infants > one month to one year	95 (42.3)	71 (46.3)	89 (57.5)	255(47.9)	P < 0.01
Pre-school age > one year to four years	49 (22.1)	41 (27.9)	23 (15.0)	113 (21.6)	
School age > four years	78 (35.1)	35 (23.8)	41 (26.8)	154 (29.5)	
Total (%)	222 (42.5)	147 (28.2)	153 (29.3)	522 (100)	
Sex					
Men (%)	113 (50.9)	81 (55.1)	91 (59.5)	285 (54.6)	P = 0.3
Women (%)	109(49.1)	66 (44.9)	62 (40.5)	237 (45.4)	
Total (%)	222 (42.5)	147 (28.2)	153 (29.3)	522	

Baseline characteristics	25(OH)D status				
Age group	Sufficient, n (%)	Insufficient, n (%)	Deficient, n (%)	Total	P-value
Infants > one month to one year	95 (42.3)	71 (46.3)	89 (57.5)	255 (47.9)	P < 0.01
Pre-school age > one year to four years	49 (22.1)	41 (27.9)	23 (15.0)	113 (21.6)	
School age > four years	78 (35.1)	35 (23.8)	41 (26.8)	154 (29.5)	
Total (%)	222 (42.5)	147 (28.2)	153 (29.3)	522 (100)	
Sex					
Men (%)	113 (50.9)	81 (55.1)	91 (59.5)	285 (54.6)	P = 0.3
Women (%)	109(49.1)	66 (44.9)	62 (40.5)	237 (45.4)	
Total (%)	222 (42.5)	147 (28.2)	153 (29.3)	522	

There was a significant difference in the mean level of vitamin D among survivors vs. non-survivors (P ≤ 0.01) and septic vs. non-septic cases (P ≤ 0.01) (Table 2). Of 522 patients, 435 (83.3%) children survived and 16.7% died. Combined, 66.6% of the total deaths had low 25(OH)D levels (deficient = 37.9%; insufficient = 28.7%), and 29 deaths (33%) were associated with normal 25(OH)D levels. Mortality was higher in children with 25(OH)D deficiency (P = 0.03; Table 3).

**Table 2:** Mean 25(OH)D levels in survivors vs. non-survivors and sepsis vs. non-sepsis SD, standard deviation

Variables	Outcome	Mean	SD	P-value
25(OH)D levels	Survivors	32.61	23.77	P < 0.01
	Non-survivors	26.51	16.37	
	Sepsis			
	No	35.69	25.31	
	Yes	25.60	16.83	

**Table 3:** Association of 25(OH)D status with mortality, sepsis, and culture positivity

Mortality	25(OH)D status				P-value
Outcome	Sufficient, n (%)	Insufficient, n (%)	Deficient, n (%)	Total	P = 0.03
Survived	193 (86.9)	122 (83)	120 (78.4)	435 (83.3)	
Death	29 (13.1)	25 (17.0)	33 (21.6)	87 (16.7)	
Total (%)	222 (42.5)	147 (28.2)	153 (29.3)	522100)	
Sepsis					
No	163 (73.4)	89 (60.5)	75 (49.0)	327 (62.6)	P ≤ 0.01
Yes	59 (26.5)	58 (39.4)	78 (51.0)	195 (37.4)	
Total (%)	222 (42.5)	147 (28.2)	153 (29.3)	522 (100)	
Culture positivity					
Negative	156 (87.2)	99 (86.8)	109 (82.6)	364 (85.6)	P = 0.47
Positive	23 (12.8)	15 (13.2)	23(17.4)	61 (14.4)	
Total	179	114	132	425	

In our study, 51% of children in the deficient group were admitted with sepsis, whereas 39.4% and 26.5% in insufficient and sufficient groups had sepsis, respectively (P < 0.01). Only 425 of 522 children's culture reports were available. Culture positivity was higher in the deficient group (17.4%) compared with the insufficient (13.2%) and sufficient (12.8%) groups (Table 3).

Of the 87 that died, 48 (55%) died due to sepsis, and the rest were due to non-septic causes. Of the 33 patients that died in the deficient group, 24 cases (72%) were due to sepsis. The mortality rate in the insufficient group was 17% (n = 25), and 48% (n = 12) of deaths were due to sepsis. A total of 39 children died in the sufficient group, of which 41.3% (n = 12) died due to sepsis and 59.7% (n = 17) died from non-septic causes (Table 4).

**Table 4:** Outcome in sepsis: deficient, insufficient, and sufficient groups

		Sepsis			
Outcome		No, n (%)		Yes, n (%)	P ≤ 0.01
Survived		288 (88.1)		147 (75.4)	
Death		39 (11.9)		48 (24.6)	
Total		327		195	
Deficient group (n = 153)					
Survived		66 (88.0)		54 (69.2)	P ≤ 0.01
Death		9 (12.0)		24 (30.8)	
Total		75		78	
Insufficient group (n = 147)					
Survived		76 (85.4)		46 (79.3)	P = 0.37
Death		13 (14.6)		12 (20.7)	
Total		89		58	
Sufficient group (n = 222)					
Survived		146 (89.6)		47 (79.7)	P = 0.07
Death		17 (10.4)		12 (20.3)	
Total, n (%)		163 (73.4)		59 (26.6)	

Multivariate analysis by logistic regression analysis was carried out to determine the independent predictors of mortality in our study population. The variants found to be significantly associated with mortality on univariate analysis were selected for multivariate analysis. Mortality is not associated with 25(OH)D deficiency or insufficiency in multilogistic regression analysis. Regression analysis showed culture positivity as an independent variable for mortality (Table 5).

**Table 5:** Multilogistic regression for mortality PEM, protein energy malnutrition

Logistic regression: mortality				
Variables	Odds ratio	95% CI for EXP(B)		P-value
		Lower	Upper	
Vitamin D insufficiency/deficiency	1.203	0.892	1.621	0.22
Culture positivity	1.939	1.034	3.636	0.03
PEM	1.435	0.867	2.375	0.16
Sepsis	1.602	0.964	2.663	0.06

**Figure 3:**
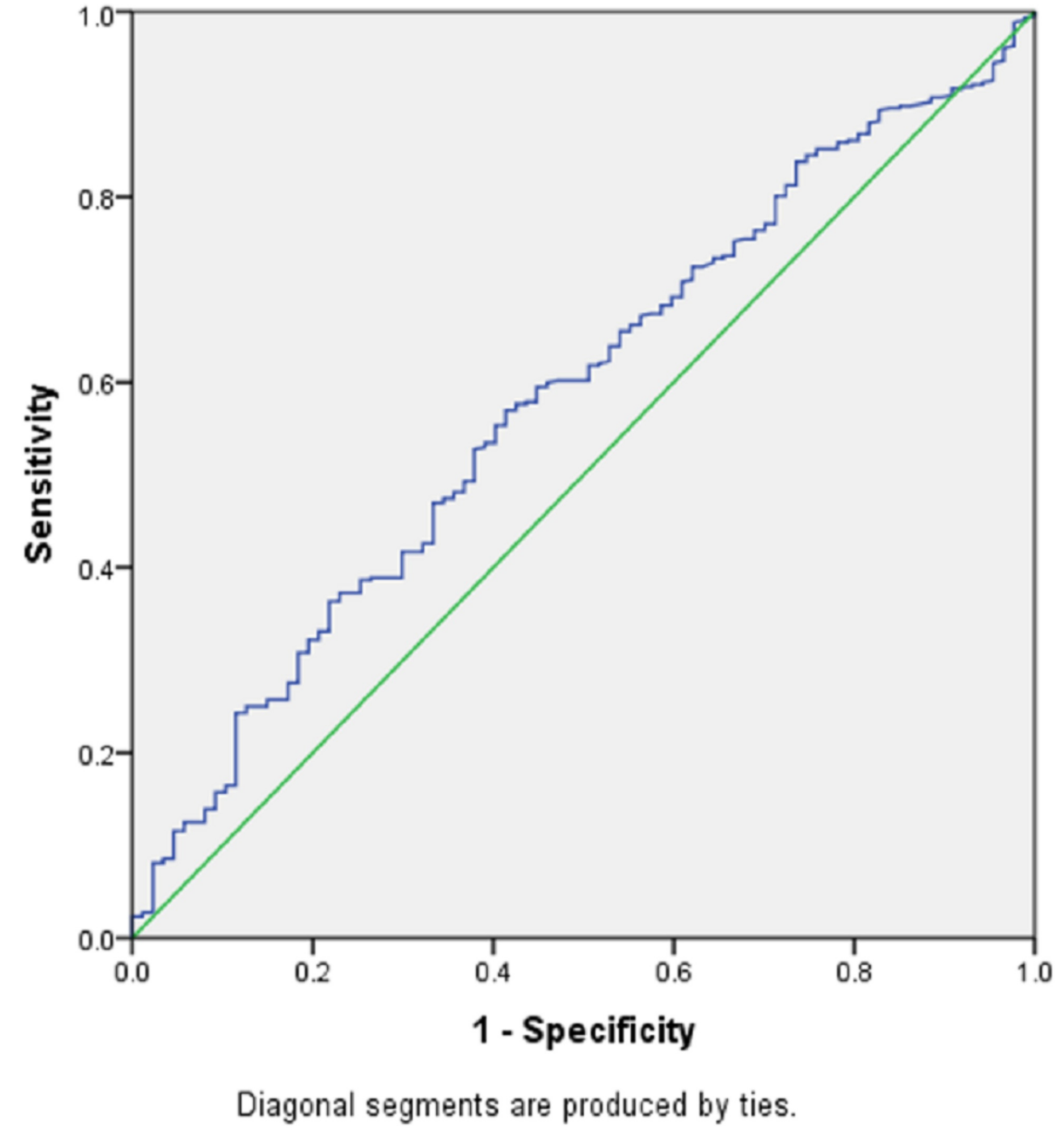
ROC curve ROC, receiver operating characteristic

The ROC curve for vitamin D levels with mortality is significant, with an area under the curve of 0.58 (P = 0.01) (Table 6, Figure 3).

**Table 6:** ROC curve: mortality vs. 25(OH)D SE, standard error; ROC, receiver operating characteristic

Area under the curve: mortality					
Test result variable(s)	Area	SE	P-value	Asymptotic 95% CI	
				Lower bound	Upper bound
25(OH)D level	0.582	0.032	0.01	0.519	0.64

 Levels of 25(OH)D at 20 ng/mL had a sensitivity of predicting the risk of mortality of 71.8%, whereas at 35 ng/mL, it was only 30.6% (Table 7).

**Table 7:** Levels of 25(OH)D predicting mortality

Area under the curve: mortality					
Test result variable(s)	Area	SE	P-value	Asymptotic 95% CI	
				Lower bound	Upper bound
25(OH)D level	0.582	0.032	0.01	0.519	0.64

## Discussion

The mean serum 25(OH)D levels in children admitted to PICUs in India is much lower (5.8 ng/mL) than what we found in our study [10]. However, this may be due to the broad range of geography and climate found in Indian, given that 25(OH)D levels depend on exposure to sunlight, climate, skin pigmentation, dietary habits, and underlying medical illness. The prevalence of vitamin D deficiency is 30% to 50% in different populations of different countries [5,11-14]. In this cross-sectional study, the prevalence of vitamin D deficiency was close to 30% in our study population. A study conducted in PICU in Delhi had reported a prevalence of 74% of vitamin D deficiency [15]. Among the studies conducted in different parts of India, the number of subjects deficient in 25(OH)D ranges from 40% to 95% [11,15,16].

Much lower serum 25(OH)D levels had been reported in pregnant women (4 ng/mL) from Turkey, and a slightly higher level (28 ng/mL) in the 1- to 11-year-old age group had been reported from the USA [11,12]. Mean 25(OH)D level was lowest in the infant age group despite children in this age group having the protection of breast milk feeding. This may suggest a case of pre-existing vitamin D deficiency in lactating mothers in India. Low 25(OH)D levels were associated with mortality in univariate analysis (P = 0.03). Mortality was higher in the 25(OH)D deficient group. Similarly, a higher incidence of death was seen in children younger than age 16 in another study, where 23.8% of patients who died were 25(OH)D deficient vs. 16.1% of those who died with sufficient 25(OH)D levels [16].

A similar high incidence of mortality in a vitamin D deficient group was also reported in adult intensive care units in the USA (24.1% in the deficient group vs. 12.2% in the insufficient group vs. 4% in the sufficient group) [4]. Also, 30-day mortality was higher in the deficient group in another study with an odds ratio of 1.85 (95% CI: 1.15-2.98; P = 0.01) [5].

The mean vitamin D level in non-survivors was 26.51 ng/mL compared with 32.61 ng/mL in survivors (P ≤ 0.01), which clearly shows a positive association of lower vitamin D level with mortality. Similar observations have been made by others. Also, in terms of all-cause mortality in adult patients in the USA, risk of death was 45% lower in those with 25(OH)D values greater than 40 ng/mL compared with those with values less than 10 ng/mL [17].

Of 33 the non-survivors in the deficient group, 77% were diagnosed as septic (P < 0.01), indicating that a higher number of non-survivors had lower vitamin D levels resulting in sepsis-related mortality. Higher 30-day mortality was reported in a lower vitamin D level group in a USA study (37% from the deficient group vs. 20% with a sufficient level) [18].

Vitamin D deficiency was strongly associated with sepsis in children according to our observation. This shows that vitamin D might be strengthening immunity, and its deficiency strongly predisposes patients to sepsis. A similar study from northern India reported that 51% of the cases admitted to the PICU had a deficient level of serum 25(OH)D [15]. Deficiency in 25(OH)D levels was also associated with increased culture positivity, though the difference was not statistically significant (P = 0.34). Our findings align with those of a prospective cohort study including 120 critically ill children in Ireland; they found that children who were deficient in serum 25(OH)D levels had a higher association with culture positivity (45% in the deficient group vs. 6% in the non-deficient group) [19].

Levels of 25(OH)D at 20 ng/mL had a 71.8% sensitivity of predicting mortality risk, whereas at 35 ng/mL, sensitivity was only 30.6%. Hence, a serum vitamin D level of 20 ng/mL can predict higher mortality with a specificity of 62.1%. A similar association was observed in an adult study where 25(OH)D levels of 10 ng/mL predicted higher mortality with a specificity of 58% [4].

There were strengths and limitations to this study. The study population was adequate for analysis. The study was conducted for one full year so that changes in weather and the effect of sunlight exposure were minimized. The study was conducted on all critically ill children to give an overall view of the prevalence of vitamin D deficiency in such imbalanced physiology and different levels of adaptive states of vital organs. Limitations of the study included its design as a prospective observational study. Because the study was conducted in a tertiary hospital, the exact picture of vitamin D status in the population and the population's vulnerability to serious illness at a community level cannot be extrapolated.

## Conclusions

25(OH)D deficiency was associated with increased culture positivity in PICU admitted cases. There was no risk of increased mortality in 25(OH)D deficiency cases. We conclude that vitamin D supplementation might be recommended for PICU admitted cases to decrease the risk of sepsis. This association can be explored more in the future among the community population for further recommendations.
